# Polaron-Adsorbate Coupling at the TiO_2_(110)-Carboxylate Interface

**DOI:** 10.1021/acs.jpclett.1c00678

**Published:** 2021-04-05

**Authors:** Alex J. Tanner, Bo Wen, Jorge Ontaneda, Yu Zhang, Ricardo Grau-Crespo, Helen H. Fielding, Annabella Selloni, Geoff Thornton

**Affiliations:** † Department of Chemistry, University College London, 20 Gordon Street, London WC1H 0AJ, United Kingdom; ‡ London Centre for Nanotechnology, University College London, 17-19 Gordon Street, London WC1H 0AH, United Kingdom; § Department of Chemistry, Princeton University, Princeton, New Jersey 08540, United States; ∥ Department of Chemistry, University of Reading, Whiteknights, Reading RG6 6AX, United Kingdom

## Abstract

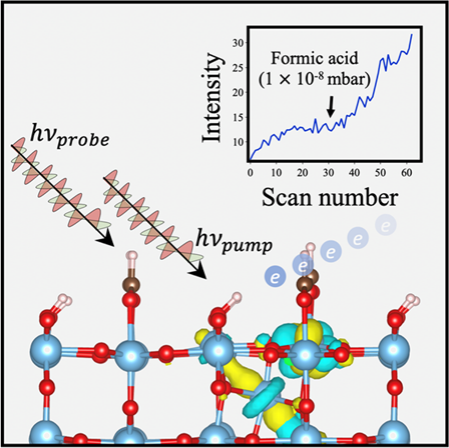

Understanding how adsorbates influence polaron behavior is of fundamental importance in describing the catalytic properties of TiO_2_. Carboxylic acids adsorb readily at TiO_2_ surfaces, yet their influence on polaronic states is unknown. Using UV photoemission spectroscopy (UPS), two-photon photoemission spectroscopy (2PPE), and density functional theory (DFT) we show that dissociative adsorption of formic and acetic acids has profound, yet different, effects on the surface density, crystal field, and photoexcitation of polarons in rutile TiO_2_(110). We also show that these variations are governed by the contrasting electrostatic properties of the acids, which impacts the extent of polaron–adsorbate coupling. The density of polarons in the surface region increases more in formate-terminated TiO_2_(110) relative to acetate. Consequently, increased coupling gives rise to new photoexcitation channels via states 3.83 eV above the Fermi level. The onset of this process is 3.45 eV, likely adding to the catalytic photoyield.

TiO_2_ is a versatile, low-cost material for a wide range of light-driven applications such as photovoltaics,^1^ water splitting,^2^ and organic photodegradation.^3−8^ It is well known that defects and their associated polarons have a large influence on the activity of these functions, behaving as charge transfer sites and electron traps.^9−11^

Carboxylic acids are ubiquitous at photocatalytic titania surfaces due to their high affinity for bonding to surface Ti atoms.^12^ Formic (HCOOH) and acetic (CH_3_COOH) acid represent the simplest carboxylic acid analogues. Their adsorption on TiO_2_ results in the formation of atomic-scale ordered overlayers at the ultrahigh vacuum (UHV), liquid and atmospheric interface, which can be observed by scanning tunneling microscopy.^12−16^ At the rutile TiO_2_(110) surface specifically, the dominant adsorption configuration of these acids consists of bidentate-bound carboxylates (RCOO^–^) at five-coordinate titanium atoms (Ti_5c_) along the [001] direction.^17^ This is accompanied by the protonation of bridging O (OH_b_) and the formation of a (2 × 1) majority phase adsorption structure (see Figure 1(a)). A minority carboxylate component is also present, which is a monodentate species oriented perpendicular to [001] and accounts for up to 1/3 of the interface.^14,18−20^ Formic and acetic acid adsorption saturates at ∼0.5 ML in UHV at 298 K, where a monolayer corresponds to the number of surface unit cells. The two terminations are denoted FA- and AA-R110, respectively.

**Figure 1:**
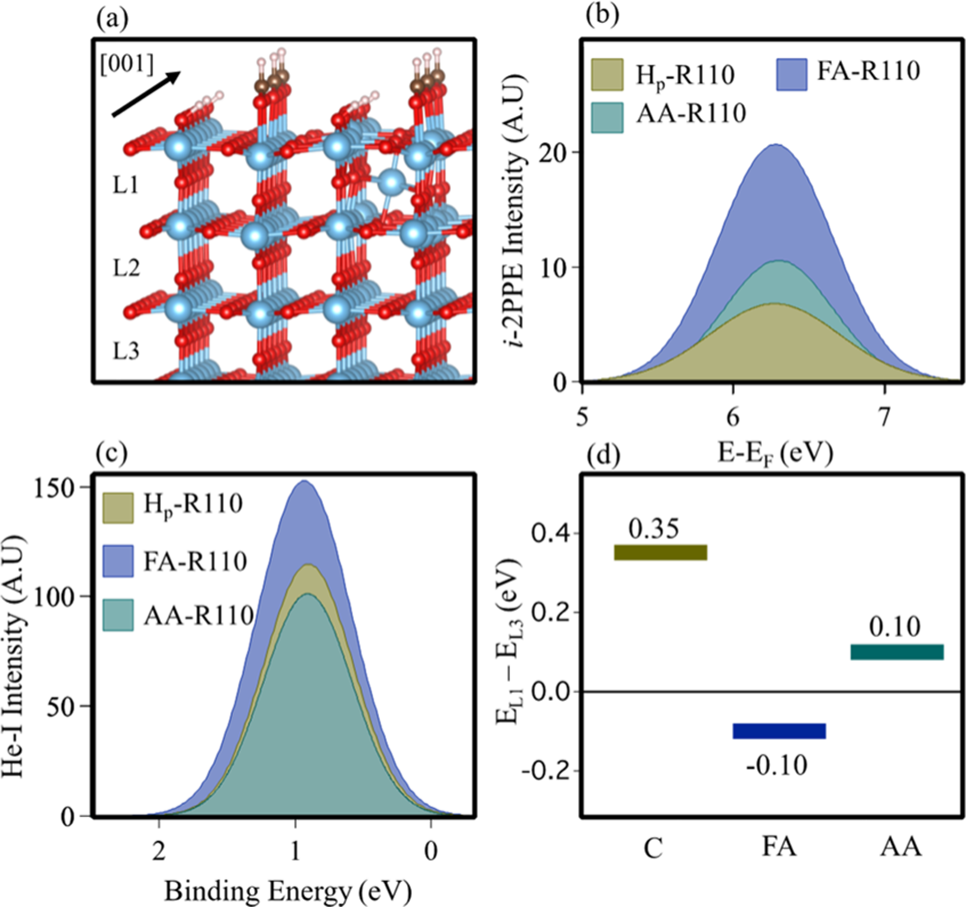
(a) Rutile TiO_2_(110) model showing the majority phase (2 × 1) formate and OH_b_ overlayer resulting from dissociative chemisorption of formic acid. An interstitial titanium atom (Ti_int_) is shown at position L1. Blue, red, brown, and white spheres represent Ti, O, C, and H, respectively. (b) Comparison of the dominant *t*_2*g*_ → *t*_2*g*_ transition in the 2PPE spectra of the H_p_-R110, FA-R110, and AA-R110 terminations at a photon energy of 3.54 eV (*p*-[001], 350 nm). Incoherent (i) features are produced according to the equation *E* – *E*_F_ = *h*ν_probe_ + *E*_intermediate_. Spectra were produced continuously at constant laser power with *in situ* gas-phase dosing. Peaks are isolated via the method described in SI Section S2. (c) Comparison of the BGS region in the UPS (He–I, 21.2 eV) spectra on the H_p_-R110, FA-R110, and AA-R110 surfaces. Peaks are isolated via the method described in SI Section S2. (d) Bar chart showing the difference in energy (Δ*E*) between a surface (L1) and bulk (L3) Ti_int_ in the clean, formate, and acetate termination of rutile TiO_2_(110), calculated with HSE06 DFT. A positive Δ*E* means that L1 is energetically less stable than L3. See details in Table S1.

Defects in rutile TiO_2_, namely surface oxygen vacancies (O_vac_) and bulk interstitial titanium atoms (Ti_int_), give rise to excess electrons in localized polaronic states.^21^ The energy levels of the electron polarons represent what are commonly referred to as the band gap states (BGS) of reduced TiO_2_, which are detectable at ∼1.0 eV binding energy (BE) in UV photoelectron spectroscopy (UPS).^22,23^ Formally, the BGS are Ti 3*d*_*xy*_ in character. This results from the Jahn–Teller splitting of Ti_3d_ atomic states in the pseudo-octahedral crystal field of rutile, which gives rise to orbitals of *t*_2*g*_- and *e*_*g*_-like symmetry.^10,24,25^ Polarons at surface O_vac_ readily react with water to form OH_b_,^9^ resulting in a small increase in the UPS BGS signal without altering the BE.^26^ This indicates that OH_b_ triggers deeper lying polarons to redistribute toward the surface, a mechanism that is also supported by density functional theory (DFT) calculations.^26,27^

Although electron polaronic states have been studied extensively, it is only recently that pump–probe studies have allowed access to their excited states. One technique employed is two-photon photoemission spectroscopy (2PPE).^28−30^ At reduced and hydroxylated TiO_2_(110) surfaces, 2PPE spectra are dominated by a *t*_2*g*_ → *t*_2*g*_ excitation feature where the excited state lies ∼2.6–2.8 eV above *E*_F_.^28,30−32^ The oscillator strength of this excitation is strongly dependent on the orientation of the electric field vector. This increases when the scattering plane is perpendicular to the [001] crystal azimuth, (*p-*[001]).^28−30,32^ In contrast, a weaker feature is observed when the scattering plane is parallel to the [001] azimuth, (*s-*[001]).^29,30^ Furthermore, it has been shown that water and methanol adsorption influences this channel, altering the orbital character and resulting in an enhancement of the *t*_2*g*_ → *t*_2*g*_ excitation oscillator strength.^27,33−35^ Despite these recent advances, the impact of carboxylates on electron polaronic states remains unknown. This understanding is potentially valuable for several technologies since carboxylates serve as the most important anchoring group for the functionalization of TiO_2_ surfaces. In this Letter, we describe a UPS, 2PPE, and DFT study that investigates the modification of electron polarons by carboxylates and their subsequent photoexcitation.

Features in 2PPE spectra are most commonly produced as a result of coherent (simultaneous two-photon excitation of an occupied state) or incoherent (two sequential one-photon excitations via an intermediate state) processes.^36^ At resonant photon energy conditions, optimal coherence between an initial and intermediate state energy results in an increase in the 2PPE intensity.^36^ In reduced and hydroxylated TiO_2_(110) (see SI for preparation methods), the resonant photon energy for the *t*_2*g*_ → *t*_2*g*_ excitation is known to be ∼3.54 eV (350 nm).^28^ In Figure 1(b), this resonance was monitored (*p*-[001], 3.54 eV, 350 nm) as a reduced rutile TiO_2_(110) sample (R-R110) partially hydroxylated in UHV (H_p_-R110, ∼0.05 ML) and was sequentially exposed to gas-phase acid. This was performed *in situ* until the saturation level (∼0.5 ML) was reached. The increase of the 2PPE resonance via hydroxylation of O_vac_ has been discussed in prior work.^27,28,30,31^ Upon creation of FA- and AA-R110, we find that the dominant incoherent process is approximately 3× and 2× larger, respectively (taken by peak area). An example of spectral evolution throughout this experiment is also shown in the Supporting Information (SI) Figure S1. In a similar framework, the BGS is monitored via UPS (He–I, 21.2 eV) and increases by a factor of ∼1.4 following formate adsorption. Following acetate adsorption the BGS area is ∼0.9 times the size, consistent with previous measurements.^37−39^ This is represented in Figure 1(c). In Figures 1(b) and (c) the peaks are isolated by removing backgrounds and are fit with Gaussian distributions (see SI, Figure S2). The difference in trend between the UPS and 2PPE data for AA-R110 is likely due to the escape depth of the two techniques (∼1 and 5 nm, for UPS and 2PPE, respectively).^40^

To further understand these observations we carried out DFT calculations (see SI for methods) using the HSE06 hybrid functional,^41^ which describes polaronic states in TiO_2_ with good accuracy.^30,32^ Ti_int_ defects were used as the source of excess electrons, and the location of Ti_int_ was varied from the immediate subsurface (L1) to two (L2) and three (L3) layers below the surface in a (4 × 2) 6-trilayers slab. Previous DFT work showed that the most stable Ti_int_ location changes upon water and methanol adsorption.^27^ Here, we determine the relative energies of Ti_int_ at clean (C) TiO_2_(110) and at the surface covered by a (2 × 1) formate or acetate monolayer. The relative stabilities of different Ti_int_ locations change significantly in the presence of a carboxylate monolayer. At the adsorbate-free surface, the most stable Ti_int_ site is L2, which is 0.46 (0.11) eV more stable than L1 (L3). In contrast, at the formate-covered surface Ti_int_ at L2 is only 0.08 eV more stable than at L1 (and 0.18 eV more stable than at L3). In the presence of an acetate monolayer, L2 and L3 are 0.09 and 0.10 eV more stable, respectively, than L1. The energetics of surface Ti_int_ following carboxylate adsorption is summarized in Figure 1(d), which shows the energy (Δ*E*) difference between surface (L1) and bulk (L3) Ti_int_ locations (see full details in SI, Table S1). Together, the UPS, 2PPE, and DFT results indicate that formate adsorption leads to the redistribution of polarons toward the surface of TiO_2_(110) through the mechanism of Ti_int_ migration. The data also show that this effect is less pronounced in the acetate termination.

On FA-R110, polaron photoexcitation was further studied by rotating the electric field vector by 90° relative to the crystal azimuth (*s-*[001]). Figure 2(a) follows the 2PPE spectrum (3.54 eV, 350 nm) as formic acid is dosed directly onto R-R110, allowing contributions from initially reactive O_vac_ (∼0.1 ML) sites to be separated.^19^ At ∼0.1 ML coverage the spectrum largely resembles that of R-R110, evidencing a slight increase in the *t*_2*g*_ → *t*_2*g*_ feature, labeled feature 1. Following saturation of the Ti_5c_ rows (∼0.5 ML), an additional feature, labeled feature 2, becomes clear. The apparent shift of feature 1 at this coverage is due to its convolution with feature 2. The inset shows the difference spectrum between the ∼0.5 ML coverage and ∼0.1 ML coverage, where the appearance of feature 2 is clear. The dependence of feature 2 on the ∼0.5 ML coverage of formate was additionally confirmed by inducing formate decomposition reactions (see SI, Figure S3).

**Figure 2:**
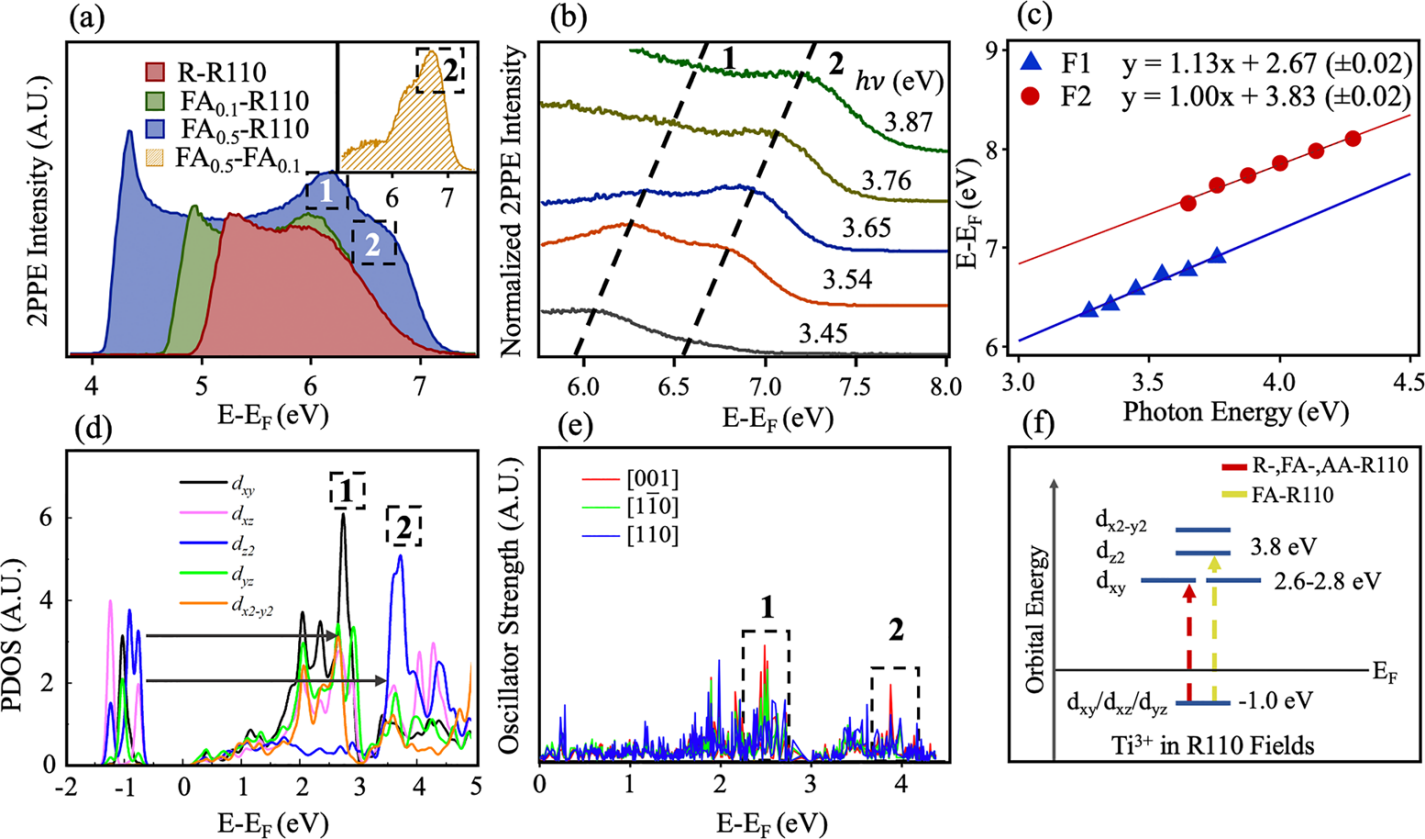
(a) 2PPE spectra (*hν* = 3.54 eV, 350 nm, *s*-[001]) of a reduced rutile TiO_2_(110) sample (R-R110) taken continuously as formic acid is dosed *in situ*. The subscript number in the legend signifies the approximate ML coverage of the formate. Numbers in dashed boxes represent the position and label of the respective features. Feature 1 denotes the previously identified *t*_2*g*_ → *t*_2*g*_ transition. The inset shows the spectrum with 0.5 ML coverage minus that at 0.1 ML coverage. (b) 2PPE spectra of selected regions containing features 1 and 2, with photon energies (*hν* = 3.44–3.87 eV, 360–320 nm), *s*-[001]). Spectra are normalized to fit the figure window for clarity. (c) Plot of the photon energy dependence of the two fitted peaks in FA_0.5_-R110 (see Section S2 in the SI for the fitting procedure) given by the equation for the incoherent process, *E* – *E*_F_ = *h*ν_probe_ + *E*_intermediate_. (d) Computed PDOS of Ti^3+^ states on FA-R110, with Ti_int_ located at L1. Peaks in the conduction band are labeled to correspond to features 1 and 2. (e) Computed oscillator strengths for transitions from BGS to the conduction band in the same system considered in (d). Red [001], green [110], and blue [110] represent directions of transition dipole moments. Peaks in the oscillator strengths are labeled that coincide with features 1 and 2. (f) Scheme showing the transitions of features 1 and 2. Feature 2 represents a *t*_2*g*_ → *e_g_* excitation. A red arrow represents transitions observed in R-, FA-, and AA-R110. A yellow arrow represents transitions observed in FA-R110 only.

The photon energy dependence of feature 2 was also examined and is shown in Figure 2(b). Feature 2 occurs close to the *E*_F_ + 2*hν* maxima of the 2PPE spectra and has an onset *hν* of ∼3.45 eV (360 nm). 2PPE spectra with *hν* > 3.54 eV (350 nm) show that feature 2 becomes more prominent in the spectra as feature 1 is less resonant. It is also observed that feature 2 is visible at much higher photon energies compared to feature 1. Figure 2(c) shows the plot of final-state energy (*E* – *E*_F_) versus photon energy (eV). It is well understood that in these plots incoherent and coherent processes produce gradients of 1 and 2, respectively.^36^ Both features are produced via an incoherent process where the excited state lies ∼2.7 and 3.8 eV above *E*_F_ for features 1 and 2, respectively (given by the *y*-intercepts). In both plots, photon energies are chosen so as to minimize overlap of the features.

DFT is again used to obtain insight into the origin of the observations. Figure 2(d) shows the partial density of states (PDOS) of excess electrons from Ti_int_ at L1 of a TiO_2_(110) surface with a (2 × 1) formate overlayer. The distribution has been separated into individual *d*-orbital contributions. The excited state energies of features 1 and 2 are represented clearly by significant density of states in the Ti^3+^ conduction band having *d*_*xy*_ and *d*_*z*_^2^ orbital character, respectively. Figure 2(e) shows the results of associated oscillator strength calculations for BGS to conduction band excitation. Peaks corresponding to both features are observed. The transition dipole moment for feature 1 lies in both the [001] and [11̅0] direction in this environment. In contrast, a transition dipole moment for feature 2 is present only in the [001] direction, explaining the observed polarization dependence. Features 1 and 2 therefore represent an excitation from occupied states *t*_*2g*_-like in character to unoccupied states of *t*_*2g*_- and *e*_*g*_-like character, respectively. A schematic of this excitation scheme is shown in Figure 2(f). Extended PDOS and oscillator strength calculations (Ti_int_ L1–L3) showing the effects of carboxylates on polaron orbital energies are given in the Figure S4.

Adsorption of the carboxylates leads to a pronounced reduction in the workfunction (5.1, 4.4, and 4.2 eV for R-, FA-, and AA-R110, respectively), which has implications for the 2PPE spectra. Specifically, this results in an enlarged 2PPE spectral window and an increased scope to study lower energy photoexcitation processes. However, at higher photon energies lower energy 2PPE features are often imperceptible due to dominating coherent valence band contributions, as well as single-photon photoemission from states near *E*_F_. Figure 3(a) shows the 2PPE spectra of AA-R110 (*p-*[001], 3.75–3.35 eV, 330–370 nm). As expected in this orientation, a strong peak associated with feature 1 is present. At ∼5.2 eV above *E*_F_ a broad feature is present that is unaffected by the shifting photon energy. This feature is also present in the 2PPE spectra (*p-*[001]) of FA-R110 (see Figure S5). We assign this distribution to Auger electrons, ejected from the BGS via the multiphoton excitation and recombination of valence band electrons. This feature also acts as a normalization point. The Auger feature is discussed in further detail inFigure S6.

**Figure 3:**
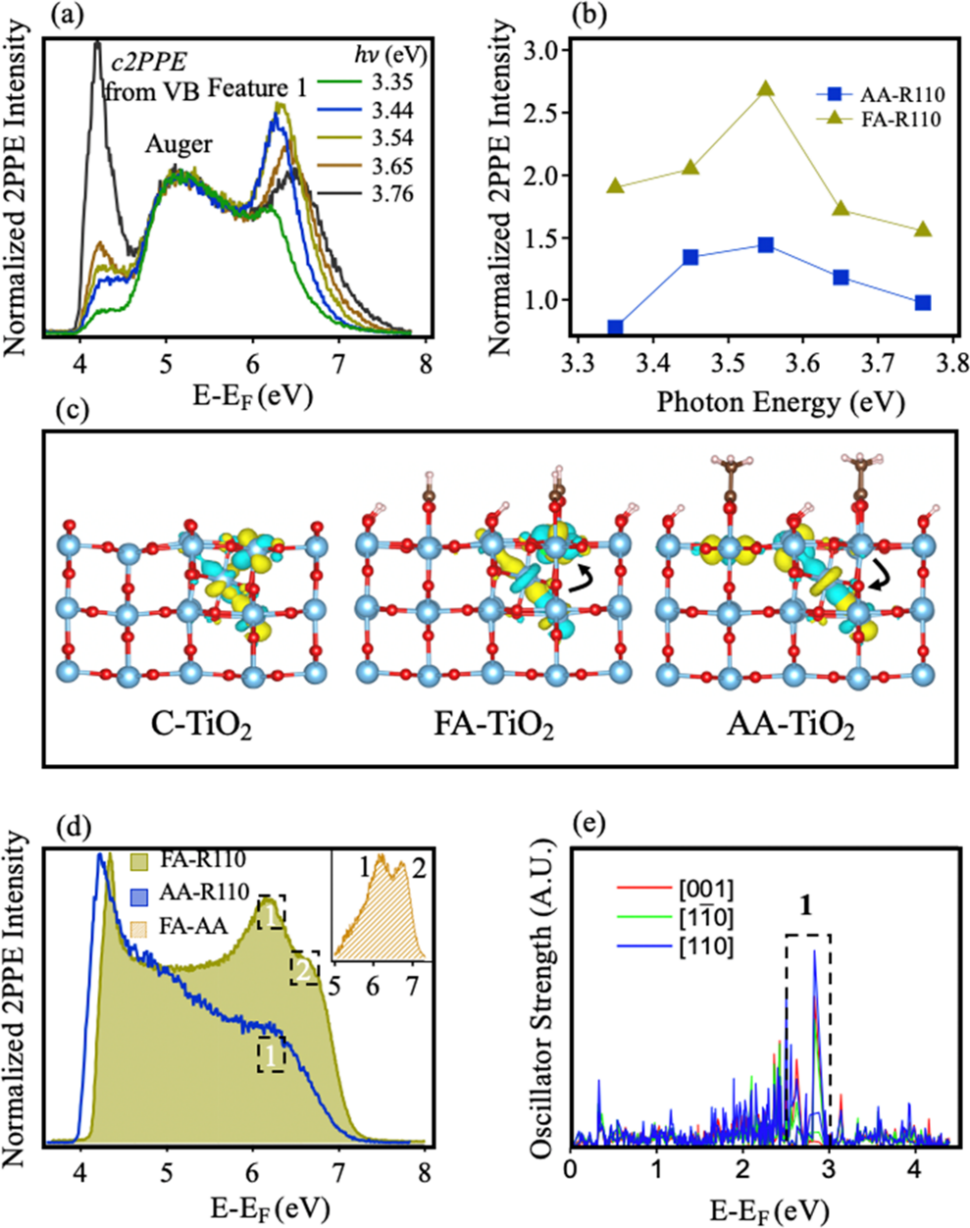
(a) 2PPE spectra (*hν* = 3.35–3.75 eV, 370–330 nm, *p-*[001]) measured from AA-R110. Three features corresponding to feature 1, Auger electrons, and valence band coherent 2PPE (c2PPE) from TiO_2_ are labeled. Spectra are normalized to the Auger feature peak intensity. (b) Peak intensity of feature 1 between (*hν* = 3.35–3.75 eV, 370–330 nm, *p-*[001]), normalized to the Auger feature, for both FA-R110 and AA-R110. (c) Spin density contour of BGS for Ti_int_ at the L1 site in the C-R110, FA-R110, and AA-R110 terminations. Arrows showing electron transfer to represent the relative attractive and repulsive properties of acid adsorbates relative to the clean surface. The red, light blue, brown, and pink spheres represent Ti, O, C, and H atoms, respectively. (d) Comparison of FA-R110 and AA-R110 2PPE (3.54 eV photons, 350 nm, *s-*[001]). The inset shows the difference spectrum between the two terminations. (e) Calculated oscillator strengths from BGS to the conduction band with L1 Ti_int_ in the acetate-terminated system. Red [001], green [110], and blue [110] represent directions of the transition dipole moments.

Figure 3(b) compares the wavelength-dependent intensity of feature 1 in FA- and AA-R110 (*p-*[001]). This comparison was made by normalizing to the Auger feature, which is present in both terminations. FA-R110 has an increased intensity of feature 1 relative to AA-R110 in all wavelengths tested. Furthermore, in both terminations 2PPE with *hν* = 3.54 eV (350 nm) produces the most intense peak, as for the adsorbate-free surface. This demonstrates that there is no distinct adsorbate-induced splitting of the occupied and unoccupied *t*_2*g*_ orbitals undergoing excitation.

There are a number of potential reasons for the differences in spectral intensity for the two adsorbates, with Ti_int_ migration and photoelectron attenuation important factors. However, DFT results suggest an additional important element. Due to the electron-donating effects of the methyl substituent, acetate repels excess electrons from the adsorbate. This is in contrast to formate, which attracts them. This is evidenced in Figure 3(c), where the spin density contour of four distinctly located excess electrons in C-, FA-, and AA-R110 are shown. Further modifications by the adsorbates can also be seen in this model. Specifically, in C-R110 the occupied states contain only orbitals of *t*_2*g*_-like character. However, following adsorption of FA and AA, new orbital characters arise. Focusing on the excess electron localized at Ti_int_ in FA- and AA-R110, a *d*_*z*^2^_-like orbital character can be identified. This change can be understood as an adsorbate-induced local crystal field. Specifically, the original octahedral crystal field is tilted into a trigonal prismatic field. In this new field, *d*_*z*^2^_ orbitals are lower in energy than the other 3d orbitals and subsequently appear in the spin density contour (Figure 3(c)) and PDOS (Figure 2(d)) (see also Figure S4). The density of those electrons in a trigonal prismatic field is governed by the electronegativity of the acid. In FA-R110, electrons are attracted away from Ti_int_, resulting in a higher proportion of surface localized *t*_2*g*_-like states compared to AA-R110. These surface states can undergo additional couplings between *t*_2*g*_ and *d*_*z*^2^_, which result in the appearance of feature 2 in the 2PPE spectra of FA-R110 and its absence in AA-R110. This comparison is shown in Figure 3(d) (*s-*[001], 3.54 eV, 350 nm, see Figure S7 for further AA-R110 spectra). The absence of feature 2 in the 2PPE spectra of AA-R110 is also corroborated by the results of oscillator strength calculations in Figure 3 (e), where no clear peaks at the position of feature 2 are observed (compare Figures 2(e) and 3(e)). Furthermore, we assign feature 2 to states localized at the surface based on oscillator strength calculations, which show that feature 2 is present only in the formate termination with Ti_int_ located at L1 and L2 (see Figure S4).

In summary, we have established that the facile formation of formate and acetate overlayers has dramatic, yet differing, implications for the behavior of polaronic states in rutile TiO_2_(110). Carboxylate adsorption leads to polaron redistribution toward the surface, driven by the migration of Ti_int_. This occurs more prominently in FA-R110, compared to AA-R110. Adsorbates subsequently couple with polaronic states to form unique crystal fields which alters the orbital character. The extent of this coupling is determined by the electrostatic properties of the carboxylate. For example, at the formate termination, polarons are attracted toward the adsorbate, increasing the oscillator strength of higher energy transitions. Specifically, polarons undergo photoexcitation via an intermediate state ∼3.83 eV above *E*_F_, characterized as a *t*_2*g*_ → *e*_*g*_ transition. It is also observed that the 2PPE spectra of both carboxylate-terminated TiO_2_(110) contain significant contributions from an Auger feature. Understanding how polarons interact with adsorbates is crucial if we are to describe the role of defects in TiO_2_ catalysis. This work provides an understanding of how carboxylates may enhance the activity of polarons by increasing their density at the surface, protecting them against oxidation (see Figure S8) and giving access to alternative photoexcitation channels.

## Supporting Information Available

Experimental details, computational details, and Figures S1–S8, showing further DFT calculations and PES spectra with extended discussion (PDF)
