# Supplementary material for: Polaron-Adsorbate Coupling at the TiO2(110)-Carboxylate Interface
Source: J Phys Chem Lett. 2021 Apr 5;12(14):3571–6. doi: 10.1021/acs.jpclett.1c00678 (PMC8054240; doi:10.1021/acs.jpclett.1c00678)
Supplement: Supplementary file 1 — jz1c00678_si_001.pdf [file jz1c00678_si_001.pdf]

Supplementary Information

Polaron-Adsorbate Coupling at the TiO_2(110)-Carboxylate Interface

Alex J. Tanner[†,‡], Bo Wen[§], Jorge Ontaneda[‖], Yu Zhang[†,‡], Ricardo Grau-Crespo[‖], Helen H. Fielding[†], Annabella Selloni[§] & Geoff Thornton*[†,‡]

[†]Department of Chemistry, University College London, 20 Gordon Street, London WC1H 0AJ, United Kingdom

[‡]London Centre for Nanotechnology, University College London, 17-19 Gordon Street, London WC1H 0AH, United Kingdom

[§]Department of Chemistry, Princeton University, Princeton NJ 08540, United States

[‖]Department of Chemistry, University of Reading, Whiteknights, Reading RG6 6AX, United Kingdom.

Experimental Considerations – Photoemission Spectroscopy

Ultraviolet photoemission spectroscopy (UPS, *VG Microtech*) and two-photon photoelectron spectroscopy (2PPE) experiments were performed in a UHV system with a base pressure of $\sim 1.0 \times 10^{-10}$ mbar. The 2PPE electrons were recorded at normal emission with a hemispherical electron energy analyzer (VG Scienta R3000), with the sample biased by -6.0 V. Photoemission from the Ta sample holder was used to determine the position of E_F. UPS and 2PPE were both used to measure workfunction values. The incident angle of the laser was 68 ± 1° from the surface normal. The laser spot had a diameter of ~0.5 mm at the sample. The system is also equipped with an X-ray (*VG Microtech*) source, which enables us to perform core-level photoelectron spectroscopy (XPS). All spectra were recorded at room temperature unless otherwise indicated.

Tunable femtosecond laser pulses (280−390 nm) were generated by a Light Conversion TOPAS-c, pumped by a Coherent Legend regenerative amplifier operating at 1 kHz, seeded by a Ti-sapphire oscillator (Coherent Micra). The power was reduced to ∼1 mW using neutral density filters to minimize space-charge effects. UPS measurements were performed to ensure no laser induced defect states were created. To ensure that changes in the spectra were not due to fluctuations in laser power, the power was monitored from a separate beam via a beam splitter earlier in the optical sequence after the TOPAS-c. The polarization of light relative to the crystal orientation can be controlled in two ways: either by the use of a periscope which can be inserted at the end of the optical sequence or by rotating the crystal azimuth.

The rutile TiO_2(110) crystal was cleaned with multiple cycles of 20 min sputtering (1 keV, 1 $\mu A/cm^2$) and 10 min annealing to ∼1000 K. After cleaning, XPS spectra evidence a contamination level <0.05%. The low-energy electron diffraction pattern was sharp (1×1), being used to determine the crystal orientation. The temperature of the samples was monitored via a K-type thermocouple in close proximity to the sample, and a digital pyrometer (Minolta). Formate and acetate interfaces were produced via gas phase dosing of the acids under UHV conditions, which is well known to result in ~0.5 ML saturated surfaces. [1,2] Carboxylic acid samples were cleaned via freeze/thaw pumping to remove dissolved O_2 and CO_2, which could otherwise affect accurate monitoring of the defect states. The purity of the acids in the gas phase was monitored by a residual gas analyzer (Hiden Analytical, HAL 101). Interfaces were

characterized by XPS, which produces characteristic C 1s signatures due to the ratio of COO^- and CH_3 contributions. Partial ML coverages were characterized by XPS C 1s ratios, UPS OH 3σ ratios or 2PPE workfunction changes, depending on the experiment.

Theoretical Considerations – DFT

Spin polarized DFT calculations were conducted using the open source package CP2K. [3,4] Norm-conserved Goedecker-Teter-Hutter (GTH) pseudopotentials [5] were used to describe the interactions between ion cores and valence electrons. A molecular optimized double zeta mixed Gaussian-planewave basis set was employed to represent the electronic wavefunctions with a plane wave energy cut-off of 280 Ry. To properly describe the BGS and resonance states of excess electrons, [6] the hybrid functional of Heyd, Scuseria, and Ernzerhof (HSE06) was used for all the calculations. [7,8] Only the Γ point was sampled in reciprocal space due to the use of large supercell models. All atoms in the slab were relaxed and the convergence threshold was ~ 0.02 eV/Å.

The rutile $TiO_2(110)$ surface was modeled using slabs of six TiO_2 tri-layers and (4×2) surface supercell with dimensions (11.836 Å × 12.949 Å). As shown in Figure 1(a), Ti_{int} was introduced into location L1, L2 or L3 of the 6 tri-layers slab, respectively. The inclusion of a Ti_{int} defect results in 4 excess electrons in the system. To explore the effects of carboxylic acid adsorption, 2×1 monolayers consisting of dissociated bridging bidentate formate or acetate species were considered. Each bidentate species is bonded to two Ti_{5c} sites, while the dissociated proton is adsorbed at an O_{br} site. Oscillator strengths were calculated according to: [6]

$$\boldsymbol{f}_{\boldsymbol{cv}}^{\boldsymbol{\mu}} = \frac{2}{m_e(E_c - E_v)} |\langle v|\boldsymbol{p}_{\boldsymbol{\mu}}|c\rangle|^2$$

Here, $\boldsymbol{f}_{\boldsymbol{cv}}^{\boldsymbol{\mu}}$ is the oscillator strength in the $\overrightarrow{\boldsymbol{e}_{\boldsymbol{\mu}}}$ polarization direction, $\langle v|$ and $|c\rangle$ denote the Kohn-Sham orbitals corresponding to the BGS electrons and unoccupied MO's, respectively, E_c and E_v are the corresponding eigenvalues. $\boldsymbol{p}_{\boldsymbol{\mu}}$ is the momentum operator along $\overrightarrow{\boldsymbol{e}_{\boldsymbol{\mu}}}$. The Fermi energy E_F was defined as the conduction band minimum (CBM), in agreement with experimental observations.

List of Tables/Figures

- **Figure S1** Example of a continuous 2PPE (3.54 eV, 350 nm) experiment involving formic acid dosing onto a rutile $TiO_2(110)$ sample.

- **Table S1** Energies of Ti_{int} in different layers (L1, L2, L3) of $TiO_2(110)$ with carboxylate adsorbates relative to the pristine surface.

- **Figure S2** Example of normalization and fitting procedure of UPS and 2PPE spectra.

- **Figure S3** Example of a continuous 2PPE (3.76 eV, 330 nm) experiment involving the heating of a formate saturated rutile $TiO_2(110)$ sample to remove feature 2.

- **Figure S4** PDOS and oscillator strengths for Ti_{int} located at L1, L2 and L3 at the clean, formate and acetate terminations.

- **Table S2** Surface induced broadening of BGS.

- **Figure S5** Further 2PPE spectra of FA-R110 (*p*-[001]).

- **Figure S6** Auger features in 2PPE Spectra - Discussion and Extension of Figure 3(a), 2PPE measurements of the acetate terminated $TiO_2(110)$ surface with higher energy photons.

- **Figure S7** Further 2PPE spectra of AA-R110 (*s*-[001]).

- **Figure S8** Discussion and example of a continuous 2PPE (3.54 eV, 350 nm) experiment involving O_2 dosing onto a formate terminated $TiO_2(110)$ sample.

S1 - Example of a continuous 2PPE (3.54 eV, 350 nm) experiment involving formic acid dosing onto a rutile TiO_2(110) sample

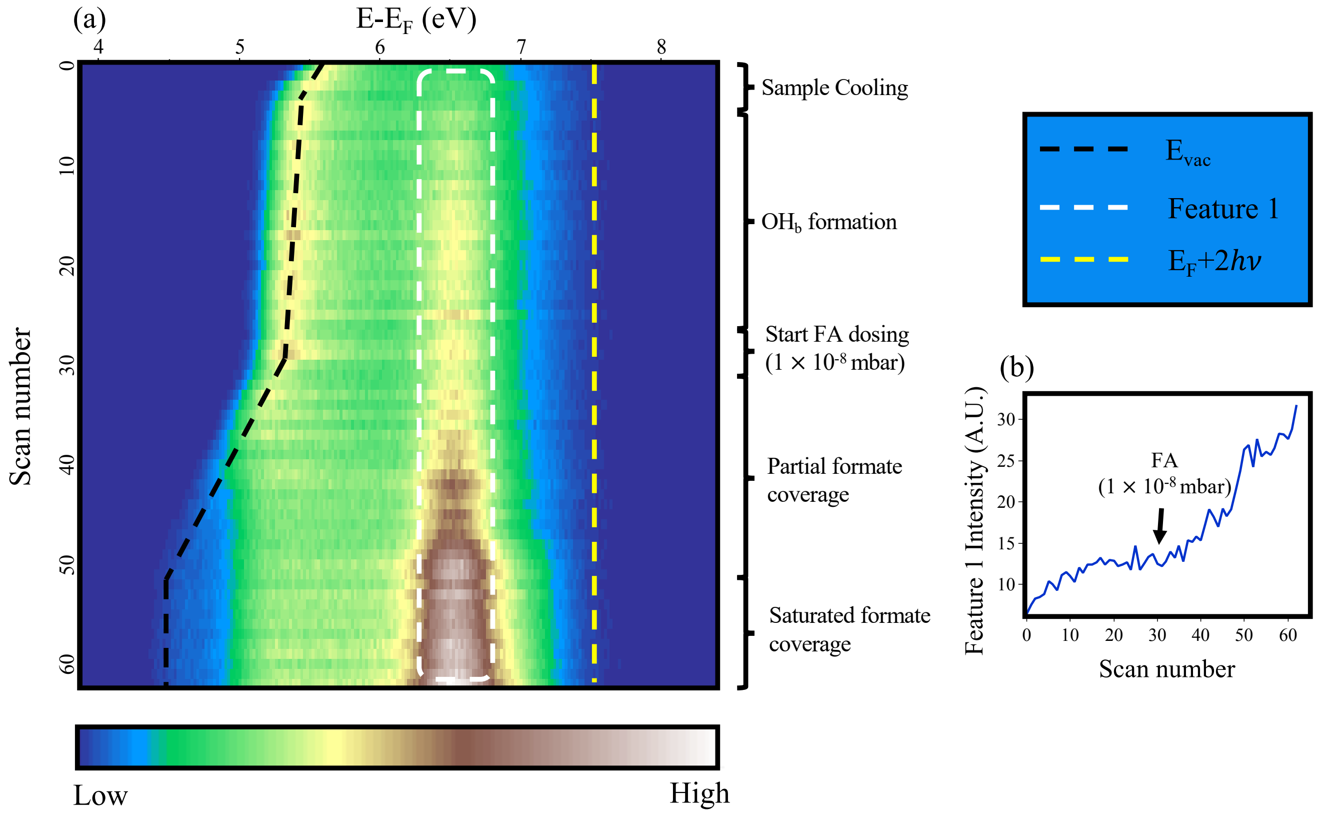

Figure S1 – (a) An example of a continuous 2PPE measurement (3.54 eV, 350 nm, p-[001])) recorded while exposing TiO_2(110) to formic acid. Black, white and yellow dashed lines represent the positions of E_{vac}, feature 1 and $E_F + 2h\nu$, respectively. **(b)** The changing intensity of feature 1 throughout this experiment.

Table S1 - Relative energies of Ti_{int} in different subsurface layers (L1, L2, L3) of the clean (C-R110), formate/OH covered (FA-R110) and acetate/OH covered (AA-R110) surfaces (see Figure 1(a)). On each surface, energies are referenced to that of the corresponding L1 defect. (Unit: eV)

	**L1**	**L2**	**L3**
**C-R110**	0.0	-0.46	-0.35
**FA-R110**	0.0	-0.08	0.10
**AA-R110**	0.0	-0.09	-0.10

S2 - Example of normalization and fitting procedure in UPS and 2PPE spectra

Normalization of 2PPE spectra

In previous work, the comparison of TiO_2 2PPE spectra across experiments is achieved by normalizing at the workfunction cut-off energy. [9-11] Normalization is required principally because of fluctuations in laser power typical of femtosecond laser systems or when changing the photon energy. Taking this approach assumes that the 2PPE signal at the cut-off remains constant. However, this assumption becomes unreliable when the workfunction (and therefore cut off energy) changes. We address this issue in two ways. Firstly, in Figures 1(b,c) and Figure 2(a), where the workfunction changes significantly, we take measurements *in-situ* to reduce fluctuations in laser power. Secondly, in Figures 3(a,b) and Figures S5,6, where the workfunction is constant, we identify an Auger feature that acts as an ideal normalization point (see S6 for further details).

Fitting of 2PPE spectra

The fitting of TiO_2 2PPE spectra has typically been achieved with Gaussian or Voigt profiles with Tougaard or linear backgrounds. [9,12,13] When using peak fitting to isolate peaks (Figures 1(b) and 2(c)) we apply Tougaard, linear and exponentially modified Gaussian backgrounds to determine the energy and intensities of our peaks. We restrict peak fitting to features far removed from the background low energy cut-off, where the background signal is minimal.

Background removal in UPS spectra

The background removal for UPS spectra employed a Tougaard function. This is a well-established method that describes the secondary electron contribution by a calculated loss-function.[14]

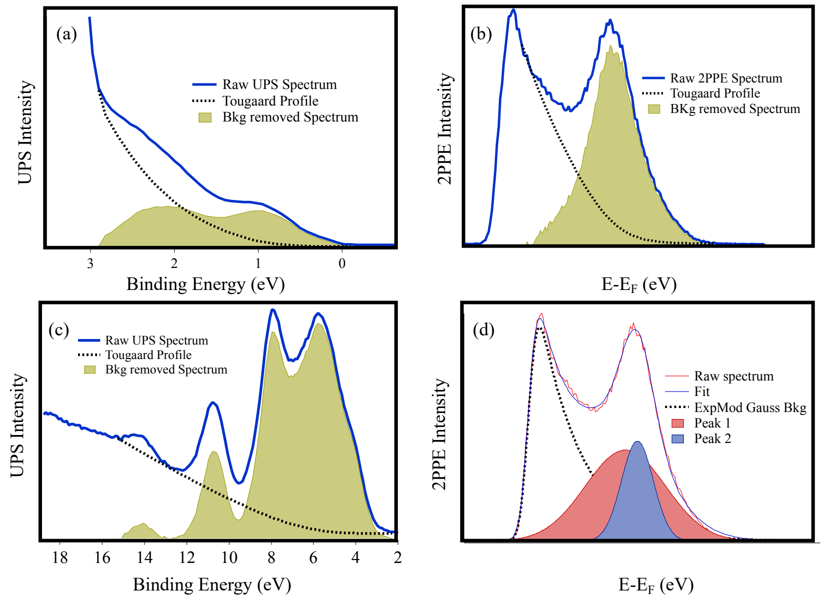

Figure S2 –(a) Examples of He-I (21.2 eV) spectra from a reduced TiO_2(110) (R-R110) surface. The peak at approx. 2.3 eV binding energy arises from a He-I satellite contribution. **(b)** Example of a 2PPE spectrum of R-R110 with a Tougaard profile removed. The remaining spectrum can then be analyzed. **(c)** Example of He-II (40.8 eV) spectrum of FA-R110 with Tougaard profile removed, leaving the remaining molecular orbital features. **(d)** 2PPE spectrum of R-R110 with an exponentially modified Gaussian distribution as a 2PPE background. In 2PPE, secondary electrons have different contributions depending on the workfunction, photon energy and material. A range of background techniques is applied including Tougaard [14], Linear [13] and exponentially modified Gaussian (shown in (d)) to accurately determine the peak location.

S3 - Formate decomposition on rutile TiO_2 (110)-monitored by 2PPE

To confirm the dependence of feature 2 on the formate overlayer, 2PPE measurements were recorded from the formate saturated surface as it was heated. At ~340 K, an increase in the workfunction indicates the disruption of the formate overlayer, which is accompanied by the sharp disappearance of feature 2. By 385 K, the workfunction value reaches a plateau at ~5.1 eV, which is close to the value of the freshly prepared reduced surface, indicating that both the majority and minority species are disrupted by the heating. Upon cooling, the workfunction decreases slightly by 0.1 eV and feature 2 does not reappear.

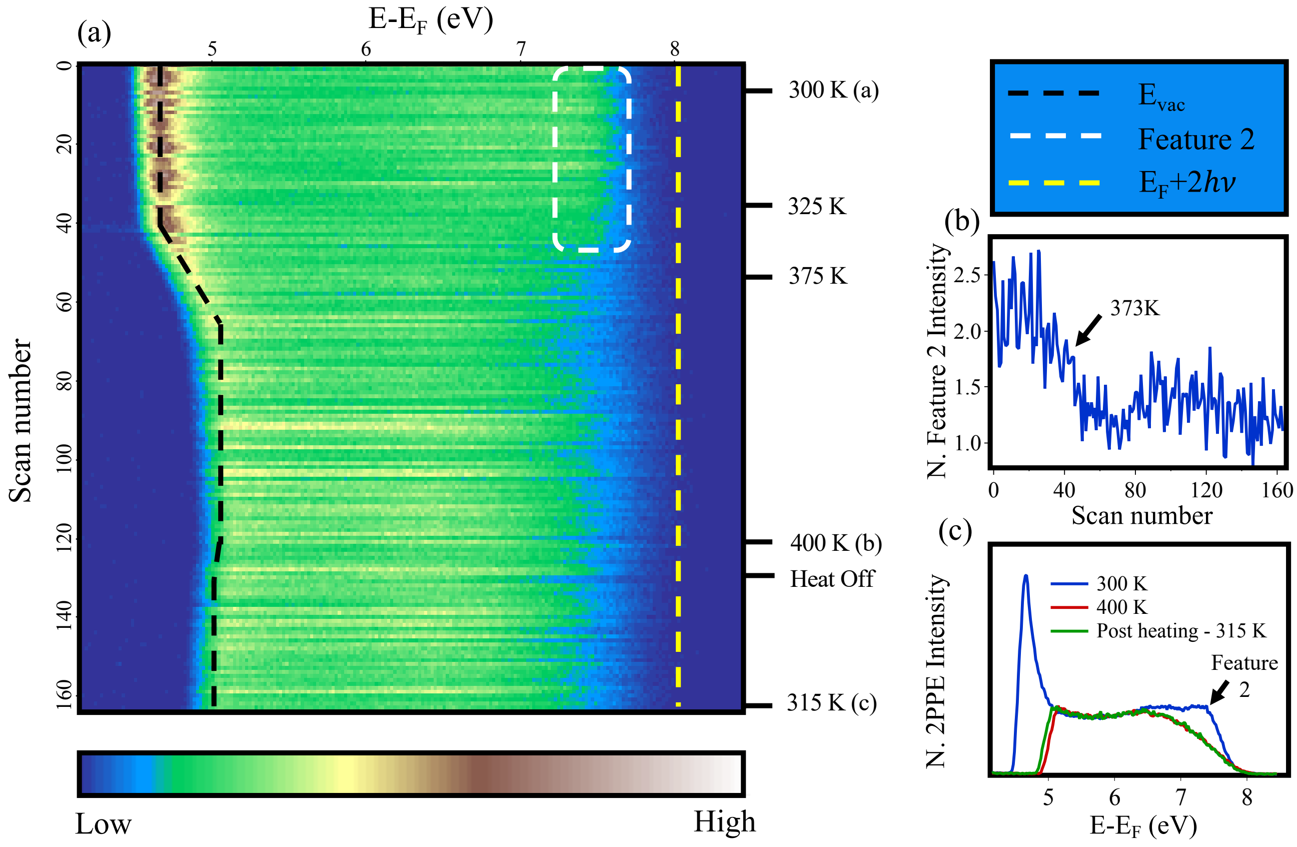

Figure S3 – (a) Continuous 2PPE measurement (3.76 eV, 330 nm, *s*-[001])) of the formate saturated $TiO_2(110)$ interface whilst heating. Heating was achieved via thermal conduction of a filament located behind the sample. Workfunction change and decrease in feature 2 starts to occur at approximately 340 K and reaches a constant by 385 K. Temperature is recorded by a K-type thermocouple adjacent to the sample. Black, white and yellow dashed lines represent the positions of E_{vac}, feature 2 and $E_F + 2h\nu$, respectively. **(b)** Changing intensity of feature 2 throughout this experiment, normalized (N.) to the intensity at 5.2 eV ($E-E_F$). **(c)** Normalized (N.) 2PPE spectra at points (a, 300 K), (b, 400 K) and (c, 315 K post heating). Normalized at 5.2 eV ($E-E_F$).

S4 - PDOS and oscillator strengths for Ti_{int} located at L1, L2 and L3 at formate and acetate terminated rutile TiO_2(110).

Effects of Ti_{int} and adsorbed carboxylates on the crystal field

At rutile TiO_2(110) surfaces, the calculated oscillator strength of the feature 1 transition is enhanced when the surface contains hydroxyls. This is due to the occupied t_{2g} state acquiring more pronounced d_{xy} character, and thus coupling more effectively to the Ti^{3+} resonant excited states in the conduction band (CB). [6,15] This trend is maintained on formate and acetate covered rutile TiO_2(110) surfaces. However, the presence of carboxylates has a distinct impact on excess electrons at Ti_{int}. As can be seen from the PDOS, occupied states of d_{z2} character appear in carboxylate terminated models. The spin density contour shows that these d_{z2}-like states are solely located at the Ti_{int} site, indicating that those electrons are more appropriately described according to a trigonal prismatic crystal field rather than the usual octahedral field of Ti ions in TiO_2. On the other hand, electrons at a larger distance from the interstitial remain in an octahedral field and occupy orbitals of t_{2g}-like symmetry. This gives rise to a complex mixture of states induced by the adsorbate. The proportion of electrons present in each crystal field is dependent on the properties of the specific carboxylate. Formate attracts excess electrons towards it, meaning there is a higher proportion of electrons away from Ti_{int}, and therefore more electrons in an octahedral field. This is evidenced in the PDOS by an increased density of states with d_{xz} character. Oscillator strength calculations suggest that these states couple with d_{z2} orbitals and give rise to feature 2 in the 2PPE spectra. This also explains the absence of feature 2 in the acetate terminated models, where there is an increased density of occupied d_{z2} states.

Effects on the distribution of gap states

Table S2 Energy difference between the highest and lowest BGS peak in the PDOS of Ti_{int} in layers 1-3 of pristine TiO_2(110) (C-R110), the formate/OH covered surface (FA-R110) and acetate/OH covered surface (AA-R110). (Unit: eV)

	**L1**	**L2**	**L3**
**C-R110**	0.63	0.28	0.17
**FA-R110**	0.48	0.32	0.19
**AA-R110**	0.26	0.59	0.31

The adsorbate induced surface structural distortions affect not only the excitations but also the distribution of the occupied states. As shown in Table S2, for Ti_{int} at L3, the energy distribution of the BGS is rather narrow, with all 4 states in a range 0.2-0.3 eV. For Ti_{int} at L2, all terminations experience broadening relative to L3, with BGS at the clean and formate termination separated by 0.28 and 0.32 eV, and those at the acetate termination by 0.59 eV. For Ti_{int} at both L2 and L3, carboxylate adsorption also induces a shift of the BGS peaks to slightly lower energies. Interestingly, for Ti_{int} at L1, the acetate termination shows an energy separation between the BGS of only 0.26 eV, smaller than for Ti_{int} at L3 and L2. The different behavior of this termination is likely related to the electron donating effects of the methyl group.

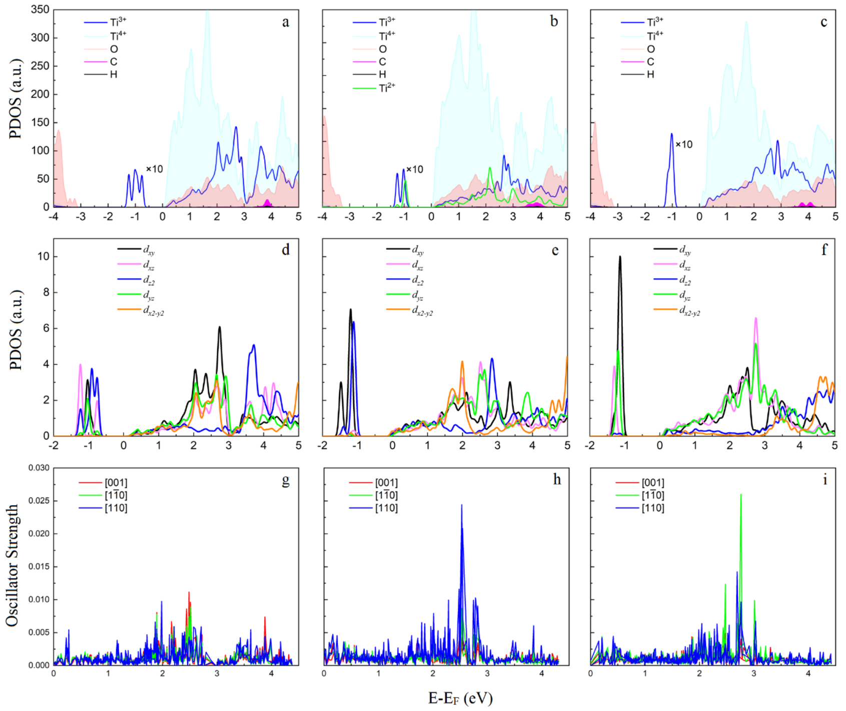

Figure S4A – Projected density of states (PDOS) and oscillator strength of 2×1 surface covered with formate/OH, with Ti_{int} located at L1 (a, d, g), L2 (b, e, h) and L3 (c, f, i), respectively. The PDOS in the upper panel were projected into different elements of the whole system, while those in the middle panel were projected into sub-d orbitals of Ti^{3+}. In regard of oscillator strength, all the contributions from BGS are added together for each case.

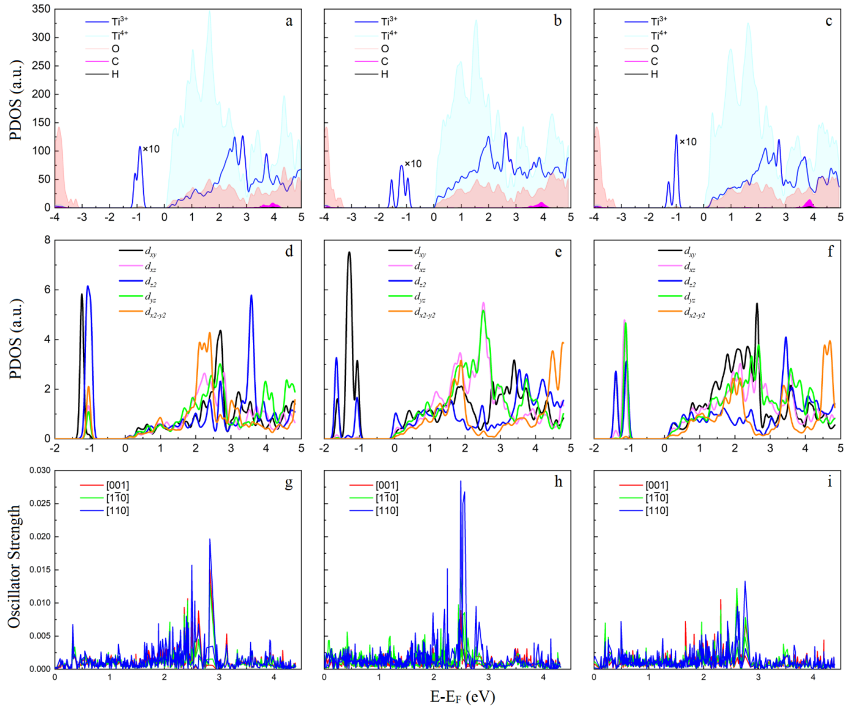

Figure S4B – Projected density of states (PDOS) and oscillator strength of 2×1 surface covered with acetate/OH, with Ti_{int} locating at L1 (a, d, g), L2 (b, e, h) and L3 (c, f, i), respectively. The PDOS in the upper panel was projected into different elements of the whole system, while those in the middle panel were projected into sub-d orbitals of Ti^{3+}. In regard of oscillator strength, all the contributions from BGS are added together for each case.

S5 - Further 2PPE spectra of FA-R110 (*p*-[001])

This figure shows further 2PPE spectra (*p*-[001], 3.35 – 3.87 eV, 370 -320 nm) of FA-R110. Four features are visible: coherent 2PPE from the valence band (VB) maximum, Auger features (see also Figures 3(a), S6), feature 1 and coherent 2PPE from the BGS. The clearer appearance of the coherent 2PPE BGS feature at higher photon energies (>3.65 eV) in FA-R110 compared with AA-R110 is in line with the UPS results in Figure 1(c). The origin of this feature can be identified from its photon energy dependence and has been reported previously. [12,13]The Auger feature has an identical distribution to that in spectra of AA-R110, making it an ideal point for normalization. There is no evidence of the feature 2 peak seen in the *s*-[001] spectra.

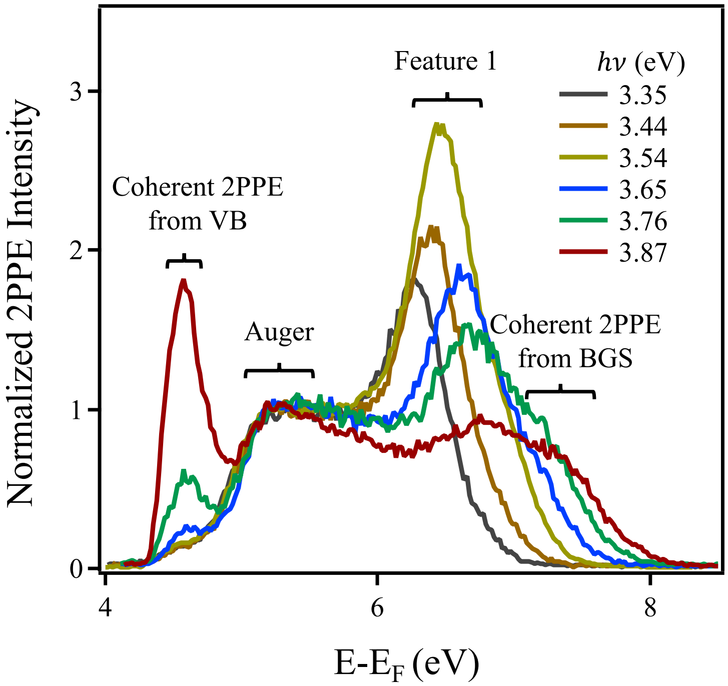

Figure S5 –2PPE measurements (3.35 – 3.87 eV, 370 - 320 nm, *p*-[001]) of the formate saturated $TiO_2(110)$ interface. As the photon energy increases, there is an increase in contribution from coherent 2PPE from the valence band tail and the appearance of the coherent 2PPE feature from the BGS. No evidence of a feature 2 peak is observed at any photon energy.

S6 - Auger electrons in the 2PPE spectra of FA- and AA-R110

In the 2PPE spectra of AA- and FA-R110 (Figures 3(a), S5, S6) there is a broad signal centered at 5.2 eV (E-E_F), that has increased intensity when light is polarized in the *p*-[001] orientation. The electron kinetic energy is unaffected by the photon energy and hence it is assigned to an Auger feature. The energy of the feature suggests a BGS origin, which would require an excitation energy of ~6.0 eV from a recombination process, given the BGS is centered at ~0.8 eV BE. A possible Auger process is therefore the ejection of BGS electrons following the multiphoton excitation and recombination of electrons from the VB to CB Ti^{4+} d-orbital states (see Figure S6, right hand side). The initial excitation must be to a state between the energy of the Ti^{3+} intermediate state (IS) and the vacuum level (E_{vac}), labeled on the figure 'Auger range'. In this picture, if VB electrons are excited within the Auger range, they can relax to a high cross-section state ~3.0 eV above E_F. If VB electrons are excited above the Auger range (exceeding the workfunction) then they are ionized, and the Auger feature is not observed. This also coincides with the dominance of the coherent 2PPE VB feature. This can be seen in Figure S6 (left hand side) at $h\nu > 4.00$ eV (310 nm), which is an extension to Figure 3(a). Although the reduction in workfunction allows for the observation of the Auger feature, previous work suggests the carboxylate overlayers themselves are the crucial factor in its appearance. 2PPE spectra with monolayer water covered TiO_2(110) does not contain an Auger feature despite the workfunction decreasing to 3.8 eV.[12,13] The possible reason for the difference between carboxylate terminated TiO_2(110) and water covered TiO_2(110) is that carboxylates facilitate the diffusion of photogenerated holes to the surface, where they are stabilized.[16-18]

The Auger feature suggests a natural position for normalization. Because these electrons arise from the recombination of a distinct state in the CB, both the relative intensity and position should be independent of the photon energy. Normalizing at this location leads to a number of observations that agree with well-established trends in the 2PPE spectra of TiO_2(110) such as the photon energy intensity dependence of feature 1 and the increase of the coherent VB feature at higher photon energies. We also note that at $h\nu > 4.00$ eV (310 nm), feature 1 is absent in the 2PPE spectra of AA-R110 and FA-R110 but present in 2PPE spectra of the clean surface.[10,13] The loss of feature 1 coincides with the dominant appearance of the coherent 2PPE VB feature, suggesting that multiphoton excited electrons in the Auger range may play a role in populating the Ti^{3+} IS at ~2.6 eV above E_F at non-resonant 2PPE conditions.

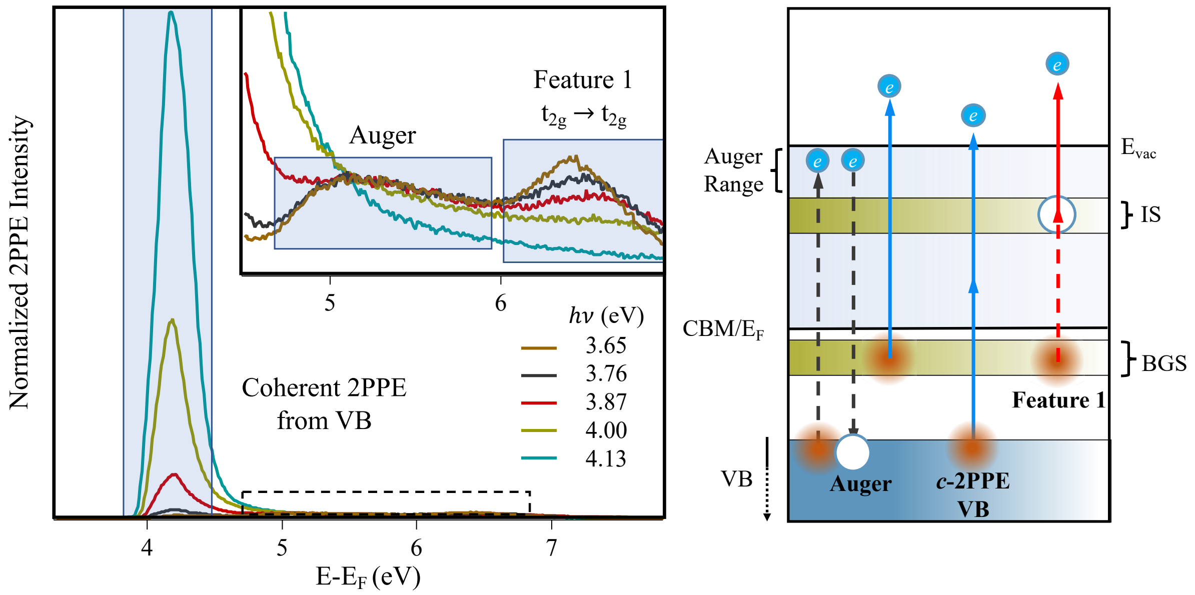

Figure S6 –Left hand side - Extension of Figure 3 (a), 2PPE measurements (3.65 – 4.13 eV, 340 - 300 nm, *p*-[001]) of the acetate saturated TiO_2(110) interface. As the photon energy increases, there is a large increase in contribution from coherent 2PPE from the valence band tail. When this coherent feature is particularly prominent, the Auger process is less pronounced, likely due to the decay process being less probable due to multiphoton photoemission. The inset shows the dashed box, expanded. Feature 1 follows the expected wavelength dependence. **Right hand side** – Schematic of processes leading to features in the 2PPE spectra of AA-R110.

S7 – Further 2PPE spectra of AA-R110 (*s*-[001])

This figure extends the AA-R110 dataset from Figure 3(d) in the main text. Feature 2 is not observed in the 2PPE spectra at any photon energy examined. Furthermore, as expected in this orientation, a weak feature 1 signal is observed.

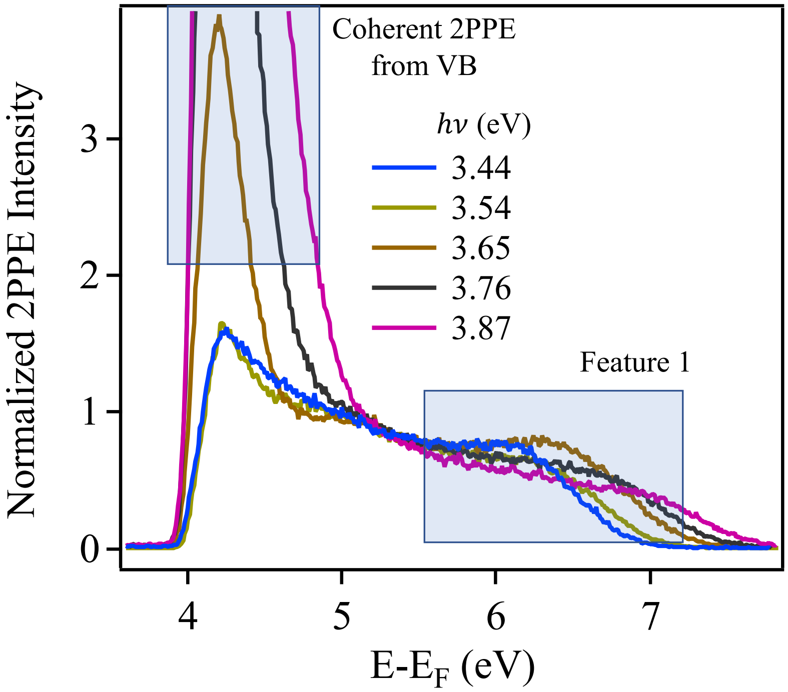

Figure S7 –2PPE measurements (3.44 – 3.87 eV, 360 - 320 nm, *s*-[001]) of the acetate saturated $TiO_2(110)$ interface. As the photon energy increases, there is a large increase in contribution from coherent 2PPE from the valence band tail. No evidence of feature 2 is observed at any photon energy.

S8 - Reactions of formate terminated rutile TiO_2 (110) with O_2

Figure S8 displays 2PPE spectra which show that a formate overlayer limits the rate of O_2 induced removal of the rutile BGS. At the H_p-R110 surface, 18 L O_2 are required for the 2PPE feature 1 signal to reach a constant bulk-related intensity that is approximately 10% intensity of the original H_p-R110 signal.[17] However, with a surface coverage of 0.5 ML formate, 1500 L of O_2 is required for the 2PPE feature 1 signal to be reduced to the intensity before exposure to formic acid. Presumably this is due to formate blocking the O_2 adsorption sites, which at the reduced/hydroxylated surface are known to be at O_{vac} and Ti_{5c} atoms. [19] This is supported spectroscopically here by three pieces of evidence. First, upon exposure of the H_p-R110 surface to O_2, there is a 0.4 eV increase of the workfunction after 1 L exposure.[17] Oxygen is known to be an electron accepting adsorbate, enhancing the surface dipole moment in the [110] direction and increasing the workfunction. In contrast, at the FA-R110 surface, there is a slow workfunction change that reaches a maximum increase of ~0.15 eV after 1500 L. The slow rate of small change in workfunction suggests that O_2 simply has very limited active adsorption sites at this termination. Secondly, no shift in the intermediate state energy is observed following O_2 dosing on this surface (1500 L), suggesting that the 2PPE signal is still arising from surface localized BGS.[17] Thirdly, there is no change in the UPS spectrum after O_2 exposure.

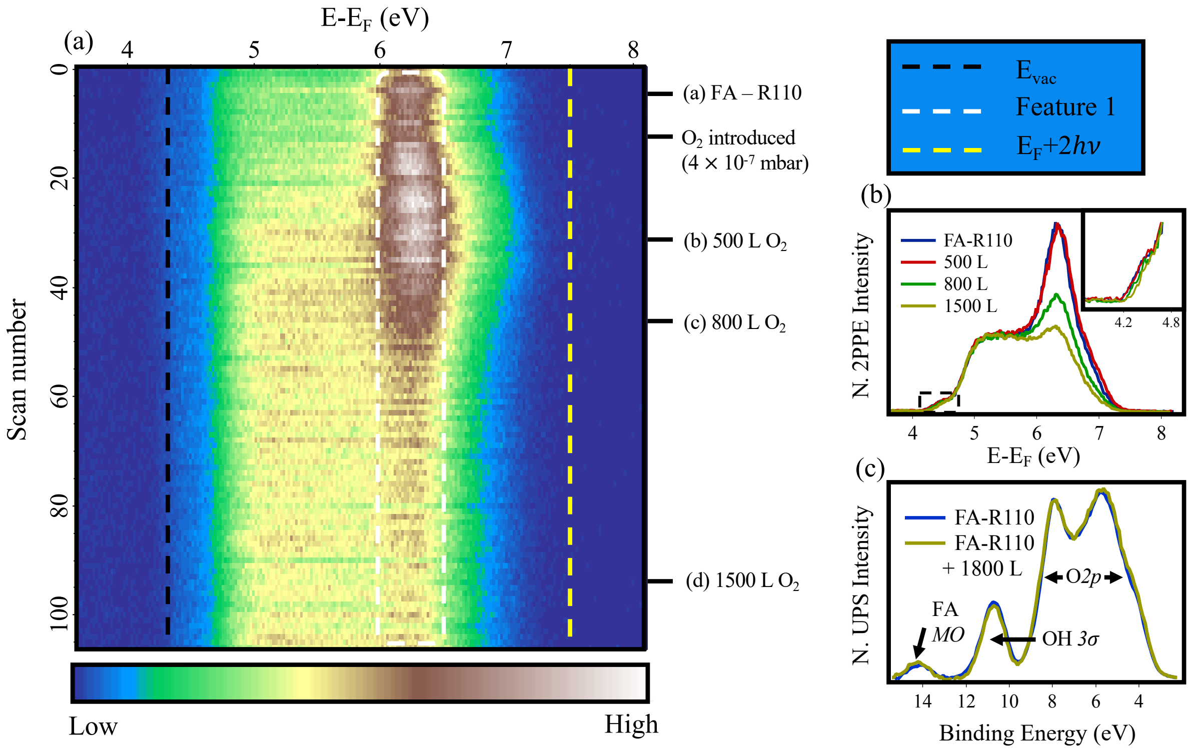

Figure S8 –(a) 2PPE measurements (3.54 eV, 350 nm, *p*-[001]) of the formate saturated TiO_2(110) interface after exposure of O_2. High exposures of O_2 are required to reduce feature 1 relative to that before exposure to formic acid. **(b)** Normalized (N.) 2PPE spectra at points (a), (b), (c) and (d), normalized to the Auger feature described in the main text. The inset shows a magnified dashed region, illustrating the small change in workfunction occasioned by exposure to O_2. **(c)** Normalized (N.) He-II (40.8 eV) spectra (Tougaard background removed) of the formate terminated TiO_2(110) surface, before and after the O_2 dosing experiment. No significant change in any features of the spectra are observed.

References

(1) Diebold, U. The Surface Science of Titanium Dioxide. *Surf. Sci. Rep.* **2003**, *48* (5-8), 53–229.

(2) Pang, C. L.; Lindsay, R.; Thornton, G. Chemical Reactions on Rutile TiO_2 (110). *Chem. Soc. Rev.* **2008**, *37* (10), 2328–2353.

(3) Kühne, T. D.; Iannuzzi, M.; Del Ben, M.; Rybkin, V. V.; Seewald, P.; Stein, F.; Laino, T.; Khaliullin, R. Z.; Schütt, O.; Schiffmann, F.; et al. CP2K: an Electronic Structure and Molecular Dynamics Software Package - Quickstep: Efficient and Accurate Electronic Structure Calculations. *J. Chem. Phys.* **2020**, *152* (19), 194103.

(4) VandeVondele, J.; Krack, M.; Mohamed, F.; Parrinello, M.; Chassaing, T.; Hutter, J. Quickstep: Fast and Accurate Density Functional Calculations Using a Mixed Gaussian and Plane Waves Approach. *Comput. Phys. Commun.* **2005**, *167* (2), 103–128.

(5) Goedecker, S.; Teter, M.; Hutter, J. Separable Dual-Space Gaussian Pseudopotentials. *Phys. Rev. B* **1996**, *54* (3), 1703–1710.

(6) Wen, B.; Hao, Q.; Yin, W.-J.; Le Zhang; Wang, Z.; Wang, T.; Zhou, C.; Selloni, A.; Yang, X.; Liu, L.-M. Electronic Structure and Photoabsorption of Ti^{3+} Ions in Reduced Anatase and Rutile TiO_2. *Phys. Chem. Chem. Phys.* **2018**, *20* (26), 17658–17665.

(7) Heyd, J.; Scuseria, G. E.; Ernzerhof, M. Hybrid Functionals Based on a Screened Coulomb Potential. *J. Chem. Phys.* **2003**, *118* (18), 8207–8215.

(8) Heyd, J.; Scuseria, G. E.; Ernzerhof, M. Hybrid Functionals Based on a Screened Coulomb Potential. *J. Chem. Phys.* **2006**, *124*, 219906. *[Erratum to Ref [7]].*

(9) Argondizzo, A.; Tan, S.; Petek, H. Resonant Two-Photon Photoemission From Ti 3d Defect States of TiO_2(110) Revisited. *J. Phys. Chem. C* **2016**, *120* (24), 12959–12966.

(10) Argondizzo, A.; Cui, X.; Wang, C.; Sun, H.; Shang, H.; Zhao, J.; Petek, H. Ultrafast Multiphoton Pump-Probe Photoemission Excitation Pathways in Rutile TiO_2 (110). *Phys. Rev. B* **2015**, *91* (15), 155429.

(11) Wang, Z.; Wen, B.; Hao, Q.; Liu, L.-M.; Zhou, C.; Mao, X.; Lang, X.; Yin, W.-J.; Dai, D.; Selloni, A.; et al. Localized Excitation of Ti^{3+} Ions in the Photoabsorption and Photocatalytic Activity of Reduced Rutile TiO_2. *J. Am. Chem. Soc.* **2015**, *137* (28), 9146–9152.

(12) Payne, D. T.; Zhang, Y.; Pang, C. L.; Fielding, H. H.; Thornton, G. Coverage-Dependent Two-Photon Photoexcitation at the H_2O/TiO_2 Interface. *Surf. Sci.* **2016**, *652* (C), 189–194.

(13) Zhang, Y.; Payne, D. T.; Pang, C. L.; Fielding, H. H.; Thornton, G. Non-Band-Gap Photoexcitation of Hydroxylated TiO_2. *J. Phys. Chem. Lett.* **2015**, *6* (17), 3391–3395.

(14) Tougaard, S. Quantitative Analysis of the Inelastic Background in Surface Electron Spectroscopy. *Surf. Int. Anal.* **1988**, *11* (9), 453–472.

(15) Wen, B.; Yin, W.-J.; Selloni, A.; Liu, L.-M. Defects, Adsorbates, and Photoactivity of Rutile TiO_2 (110): Insight by First-Principles Calculations. *J. Phys. Chem. Lett.* **2018**, *9* (18), 5281–5287.

(16) Zhang, Z.; Yates, J. T., Jr. Band Bending in Semiconductors: Chemical and Physical Consequences at Surfaces and Interfaces. *Chem. Rev.* **2012**, *112* (10), 5520–5551.

(17) Sandell, A.; Ragazzon, D.; Schaefer, A.; Farstad, M. H.; Borg, A. Photochemistry of Carboxylate on TiO_2(110) Studied with Synchrotron Radiation Photoelectron Spectroscopy. *Langmuir* **2016**, *32* (44), 11456–11464.

(18) Henderson, M. A.; White, M. J.; Uetsuka, H.; Onishi, H. Photochemical Charge Transfer and Trapping at the Interface Between an Organic Adlayer and an Oxide Semiconductor. *J. Am. Chem. Soc,* **2003**, *125* (49), 14974-14975.

(19) Wendt, S.; Sprunger, P. T.; Lira, E.; Madsen, G. K. H.; Li, Z.; Hansen, J. Ø.; Matthiesen, J.; Blekinge-Rasmussen, A.; Lægsgaard, E.; Hammer, B.; et al. The Role of Interstitial Sites in the Ti3d Defect State in the Band Gap of Titania. *Science* **2008**, *320* (5884), 1755–1759.

(20) Tanner, A. J.; Wen, B.; Zhang, Y.; Liu, L-M.; Fielding, H. H.; Selloni, A.; Thornton, G. Photoexcitation of Bulk Polarons in Rutile TiO_2, *Phys. Rev. B* **2021**, *103*, L121402.
